# Carbohydrate metabolism genes and pathways in insects: insights from the honey bee genome

**DOI:** 10.1111/j.1365-2583.2006.00677.x

**Published:** 2006-10-01

**Authors:** T Kunieda, T Fujiyuki, R Kucharski, S Foret, S A Ament, A L Toth, K Ohashi, H Takeuchi, A Kamikouchi, E Kage, M Morioka, M Beye, T Kubo, G E Robinson, R Maleszka

**Affiliations:** * Department of Biological Sciences, Graduate School of Science, The University of Tokyo Bunkyo-ku, Tokyo, Japan; † Visual Sciences and ARC Centre for the Molecular Genetics of Development, Research School of Biological Sciences, The Australian National University Canberra ACT, Australia; ‡ Neuroscience Program, University of Illinois at Urbana-Champaign IL, USA; § Program in Ecology and Evolutionary Biology, University of Illinois at Urbana-Champaign IL, USA; ¶ Department of Entomology Graduate School of Pharmaceutical Sciences, Tohoku University Aoba-ku, Sendai, Japan; ** Institute of Zoology, University of Cologne Cologne, Germany; †† Heinrich-Heine Universitaet Duesseldorf, Institut fuer Genetik Duesseldorf, Germany

**Keywords:** insect metabolome, lipid metabolism, cellulose degradation, gene synteny

## Abstract

Carbohydrate-metabolizing enzymes may have particularly interesting roles in the honey bee, *Apis mellifera*, because this social insect has an extremely carbohydrate-rich diet, and nutrition plays important roles in caste determination and socially mediated behavioural plasticity. We annotated a total of 174 genes encoding carbohydrate-metabolizing enzymes and 28 genes encoding lipid-metabolizing enzymes, based on orthology to their counterparts in the fly, *Drosophila melanogaster,* and the mosquito, *Anopheles gambiae*. We found that the number of genes for carbohydrate metabolism appears to be more evolutionarily labile than for lipid metabolism. In particular, we identified striking changes in gene number or genomic organization for genes encoding glycolytic enzymes, cellulase, glucose oxidase and glucose dehydrogenases, glucose-methanol-choline (GMC) oxidoreductases, fucosyltransferases, and lysozymes.

## Introduction

Carbohydrate- and lipid-metabolizing enzymes catalyse reactions that break down food to release stored energy and synthesize an organism's primary energy stores (reviewed by Klowden, 2002). Metabolic enzymes are typically among the most conserved genes across taxa, but the dietary specialization of the bees (Hymenoptera: Apoidea) on honey and pollen feeding may have placed selective pressure on some metabolic pathways.

The best-studied bee species, the Western honey bee *Apis mellifera* L., has colonies which are typified by a reproductive division of labour between queens and workers, and by an age-based behavioural division of labour among workers (Winston, 1987). Metabolic processes are important regulators of both caste determination and behavioural development. Caste determination occurs through differences in larval nutrition (Winston, 1987) that are independent of genetic differences: larvae fed nutrient-rich royal jelly become queens, whereas larvae that are fed a less rich diet become workers. Young worker bees specialize on a variety of tasks inside the hive including nursing, comb-building and food handling, and old workers work outside the hive as foragers for nectar and pollen. Behavioural maturation in bees is mediated by physiological changes (Robinson, 2002). For instance, lipid stores drop dramatically prior to the onset of foraging, and this has a causative role in behavioural maturation; both starvation and an inhibitor of fatty acid synthesis cause bees to begin foraging earlier in life (Schulz *et al*., 1998; Toth *et al*., 2005). In addition, many of the genes identified by a microarray study whose expression was most predictive of differences in behavioural state encode metabolic enzymes (Whitfield *et al*., 2003).

The complement of metabolic enzymes possessed by an organism may reflect its dietary specializations. Bees consume nectar and pollen as carbon and nitrogen sources, respectively, and both of these foods require extensive processing in the gut. Nectar is converted to honey by three enzymes secreted by the hypopharyngeal glands of workers. Alpha-glucosidase converts sucrose, the primary component of nectar, into glucose and fructose (Simpson *et al*., 1968; Kubo *et al*., 1996; Ohashi *et al*., 1996, 1997). Amylase hydrolyses plant starches that contaminate the nectar (Winston, 1987; Ohashi *et al*., 1999). Finally, a glucose oxidase converts glucose into gluconic acid and peroxide, both of which afford antiseptic activity to the honey (White *et al*., 1963; Winston, 1987; Ohashi *et al*., 1999). Additionally, honey bees produce a substance called propolis used in nest construction by adding secretions to plant resins (Ghisalberti, 1979; Winston, 1987). Bees are thought to have evolved from a wasp ancestor, probably a sphecid, with mouthparts capable of ingesting nectar, which began collecting pollen to feed their brood instead of hunting prey (Michener, 1974; Winston, 1987). It is possible that traces of a predatory ancestry may be observable in the profile of metabolic enzymes of the honey bee.

The sequencing of the genome of the honey bee, *Apis mellifera* (Honey Bee Genome Sequencing Consortium, 2006) enabled us to conduct genome-wide analyses of the genes encoding carbohydrate- and lipid-metabolizing enzymes, and compare these results with analysed genome sequences in the fly, *Drosophila melanogaster,* and the mosquito, *Anopheles gambiae* (Adams *et al*. 2000; Holt *et al*., 2002)*.* All three of these insects show dietary habits that in some form involve reliance on a sugar-rich substrate. Honey bees have specialized on mass storage and consumption of nectar and honey (Winston, 1987); flies consume yeast and other microorganisms on sugar-rich rotting fruit (Soliman & van Herrewege, 1988); and mosquitoes consume nectar (Gillett, 1971). Furthermore, the honey bee is known to rely strongly on the products of carbohydrate metabolism to fuel foraging flights (Neukirch, 1982; Beenakkers *et al*., 1984). Other unique characteristics associated with diet and energy requirements, such as the ability of both bees and mosquitoes to synthesize glycogen during flight (Nayar & van Handel, 1971), could lead to changes in the complement of metabolic enzymes in each of these species. Thus, we might predict enhanced differences in enzymes relating to carbohydrate metabolism when comparing orthologues of the genes encoding these enzymes across the three species.

As a first step towards understanding the honey bee metabolome and highlighting differences within the insects, we compared the complement of genes in the three species for several important pathways in energy metabolism. By placing a set of our annotated genes in the context of well-established pathways (Kanehisa & Goto, 2000), we were able to gain insight into which metabolic pathways may have undergone substantial changes during insect evolution. Our findings suggest that across these three insect species, genes coding for carbohydrate-metabolizing enzymes have undergone more changes in gene number relative to lipid-metabolizing genes. We also describe the genomic organization of several unusual carbohydrate-metabolizing enzymes with important roles in honey bee biology. These include (1) cellulase; (2) glucose oxidase and glucose dehydrogenase; (3) glucose-methanol-choline (GMC) oxidoreductases; (4) fucosyltransferases; and (5) lysozymes. Changes in gene copy number have been shown to be important sources of variation within and across species, and may act as a ‘driving force’ in phenotypic evolution (Eichler & Sankoff, 2003; Aitman *et al*., 2006). Thus, the comparisons of both the number and organization of genes encoding metabolic enzymes will give important insights into the characteristics and evolution of metabolism across insect taxa.

## Results and discussion

### Genome-wide analyses of carbohydrate and fatty acid metabolism

**Figure 1:**
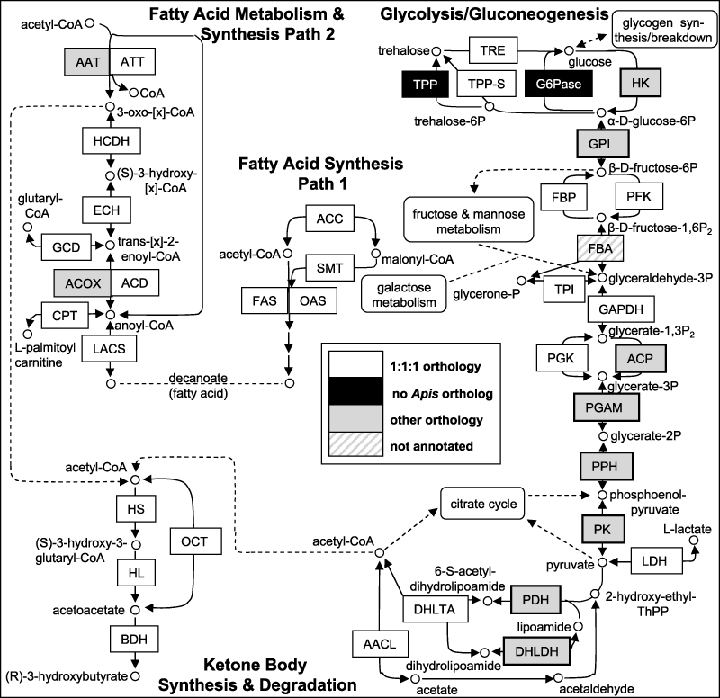
Metabolic pathways for fatty acid synthesis and metabolism, ketone body synthesis and degradation, and glycolysis/gluconeogenesis, and their relationships to the metabolism of some additional carbohydrates. Boxes are shaded according to the complements of genes in *Apis mellifera, Drosophila melanogaster* and *Anopheles gambiae.* Abbreviations: AACL, acetoacetate-CoA ligase; AAT, acetyl-CoA acetyl transferase; ACC, acetyl-CoA carboxylase; ACD, acyl-CoA dehydrogenase; ACOX, acyl-CoA oxidase; ACP, acylphosphatase; ATT, acetyl-CoA acyl transferase; BDH, 3-hydroxybutyrate dehydrogenase; CPT, carnitine O-palmitoyl transferase; DHLDH, dihydrolipoamide dehydrogenase; DHLTA, dihydrolipoamide S-acetyltransferase; ECH, enoyl-CoA hydratase; FAS, fatty acid synthase; FBA, fructose bisphosphate aldolase; FBP, fructosebisphosphatase; G6Pase, glucose-6-phosphatase; GAPDH, glyceraldehyde-3-phosphate dehydrogenase; GCD, glutaryl-CoA dehydrogenase; GPI, glucose-6-phosphate isomerase; HCDH, 3-hydroxyacyl-CoA dehydrogenase; HK, hexokinase; HL, hydroxymethylglutaryl-CoA lyase; HS, hydroxymethylglutaryl-CoA synthase; LACS, long-chain-fatty-acid-CoA ligase; LDH, L-Lactate dehydrogenase; OAS, 3-oxoacyl synthase; OCT, 3-oxoacid-CoA transferase; PDH, pyruvate dehydrogenase; PFK, 6-phosphofructokinase; PGAM, phosphoglycerate mutase; PGK, phosphoglycerate kinase; PGM, phosphoglucomutase; PK, pyruvate kinase; PPH, phosphopyruvate hydratase (enolase); SMT, S-malonyl transferase; TPI, triose-phosphate isomerase; TPP, trehalose-6-phosphate phosphatase; TPP-S, trehalose-6-phosphate phosphatase/trehalose-6-phosphate synthase fusion; TRE, trehalase.

To determine orthologous relationships of a subset of important metabolic enzymes, we first analysed pathways for fatty acid metabolism and synthesis, ketone body degradation and synthesis, and glycolysis/gluconeogenesis (Fig. 1). In general, we found a high degree of homology between species (typically > 50% identity between bee and dipteran genes), making it possible for us to predict orthology in most cases. Overall, the majority of genes found in the bee genome have simple, 1 : 1 : 1 orthology (*Apis*: *Drosophila*: *Anopheles*). However, for some enzymatic functions, we noticed changes in number in one or more of the species, and these changes seem to be more common in enzymes with roles in glycolysis and gluconeogenesis. As a rough test for the significance of these changes in gene number, we grouped enzymes into two categories: enzymatic functions with or without changes in the orthologue ratio between all three species. For this data set, there were significantly more changes in gene number for glycolytic and gluconeogenic enzymes than for enzymes in fatty acid synthesis and ketone body synthesis and degradation pathways combined (Chi-square test: d.f. = 1, chi^2^ = 5.59, *P* = 0.018).

Some enzyme types with particularly striking changes in gene number included acyl-CoA oxidase (2 : 6 : 6), acylphosphatase (2 : 6 : 2) and pyruvate kinase (2 : 6 : 1). Three glycolysis/gluconeogenesis genes have 2 : 1 : 1 orthology *–* pyruvate dehydrogenase, dihydrolipoamide dehydrogenase and phosphopyruvate hydratase – representing either recent duplications in *Apis* or gene losses in the dipterans. Finally, two enzymes found in the dipteran species, glucose-6-phosphatase and the monomeric trehalose-6-phosphate phosphatase, appear to be completely missing in the current assembly of the *Apis* genome. As glucose-6-phosphatase activity has been described in honey bee flight muscle (Surholt & Newsholme, 1981), it is possible that another phosphatase has shifted its specificity to fill this role. However, if these are true gene losses, bees would be left with a single functional pathway by which to convert gluconeogenic substrates to both of the primary carbohydrate energy stores used by insects, trehalose and glycogen.

As is evident from Fig. 1, we see more differences in gene number for glycolysis and gluconeogenesis than for fatty acid or ketone synthesis and metabolism. It is possible that some of these differences have functional consequences for the metabolism of these three insect species. Although we did not observe any gene number differences for one of the rate-limiting enzymes in glycolysis (phosphofructokinase), we did observe changes in two other enzymes with documented effects on the glycolytic flux, hexokinase and pyruvate kinase. Pyruvate kinase showed a large difference between species (2 : 6 : 1 orthology); this enzyme converts phosphoenol pyruvate to pyruvate, which may then enter the citrate cycle or other pathways. As we have no information on the expression patterns of the different pyruvate kinase genes, it is not possible to predict the functional consequences of this change in gene number. It is known that pyruvate kinase activity increases in honey bees starting a few days after emergence, and this increase has been suggested as important for carbohydrate mobilization in preparation for foraging (Harrison, 1986). In addition, the expansion of pyruvate kinase genes in *Drosophila* suggests that this enzyme has diversified, and this may allow flies to cope with different pyruvate fluxes depending on their feeding substrate, as *Drosophila* is a generalist feeder (Soliman & van Herrewege, 1988).

We saw no change in orthologue ratios for fructose bisphosphatase, considered to be the rate-limiting enzyme in gluconeogenesis. However, we found 2 : 1 : 1 orthology for three enzymes catalysing reactions involving pyruvate. The first was phosphopyruvate hydratase, which converts glycerate-2-phosphate to phosphoenolpyruvate. The other two are pyruvate dehydrogenase and dihydrolipamide dehydrogenase, which convert pyruvate to other intermediates that lead to the production of acetyl-CoA, an important metabolite that may feed into fatty acid and ketone synthesis and the citrate cycle. Although expression patterns of the *Apis* enzymes are not known, duplications in these genes could also impact the amount and localization of these metabolic intermediates in different tissues or life stages, and thus, the production of acetyl-CoA.

Taken together, these results suggest the possibility of functional differences in glycolysis and gluconeogenesis between *Apis*, *Drosophila* and *Anopheles*. Many of the differences in gene number we detected are at the early steps of glycolysis, so these differences may relate to differences in monosaccharide use or availability. Numerous differences were also found in the final steps of glycolysis; these differences suggest that there may be interspecies variation in the reliance on anaerobic energy generation. Insect flight is thought to primarily utilize very high rates of aerobic respiration, although a potential role for anaerobic respiration during short-term, high power output flight has not been fully explored (Beenakkers *et al*., 1984; Harrison & Roberts, 2000). Because of the long-distance flights made by *Apis* foragers, which have been documented at up to 14 km (Seeley, 1995), honey bees are likely to show some unique metabolic adaptations to foraging (Hrassnigg & Crailsheim, 2005). For example, honey bee workers are known to have an extremely high carbohydrate demand to fuel foraging flights (Gmeinbauer & Crailsheim, 1993) and can use starch and proline as energy sources during flight (Micheu *et al*., 2000; Hrassnigg *et al*., 2005). The foraging ‘career’ of a typical honey bee is a remarkable 800 km, after which foragers cease to replace glycogen stores in the flight muscle and die (Neukirch, 1982). Another unique aspect of honey bee energetics is the ability of honey bee workers to generate large amounts of heat through ‘shivering thermogenesis’ (Stabentheiner *et al*., 2003). This process is especially important for heat generation in clusters of winter bees, and is likely to depend on glycogen metabolism (Panzenbock & Crailsheim, 1997). More detailed functional analyses of *Apis-*specific differences in glycolysis and other pathways could uncover the enzymatic mechanisms underlying such unique aspects of honey bee foraging energetics.

We can make only limited inferences about evolution from an analysis of gene numbers alone, especially given the phylogenetic relationships between *Apis*, *Drosophila* and *Anopheles*. Studies of metabolite profiles, enzyme activities and localization patterns could shed further light on how these differences may be important for dealing with the specific energy requirements and dietary differences that characterize each insect species. Our results highlight several candidate enzymes within glycolysis and gluconeogenesis for further comparative analysis.

To determine whether the observed variation in gene number exists across all carbohydrate-metabolizing enzymes, we conducted a broader survey of *Apis* genes for enzymes involved in the metabolism of several additional sugars (a total of 32 compounds). We compiled a list of 101 genes encoding carbohydrate-metabolizing enzymes in *Drosophila* and other species (using databases at Flybase, Anobase and NCBI), and identified a total of 148 homologues for these genes in the *Apis* genome (Supplementary Material Table S1). The results indicated that there are four groups of enzymes whose gene numbers differ drastically among the three insect species: (1) cellulase, (2) glucose oxidase, (3) fucosyltransferase and (4) lysozymes (Table 1). To better understand these differences in gene number and apparent organization, we performed more detailed analyses on these four groups of enzymes along with glucose dehydrogenase and GMC oxidoreductases, from which glucose oxidase is supposed to have evolved (see below).

**Table 1:** Comparison of gene number for selected carbohydrate-metabolizing enzymes in the honey bee, *Drosophila* and *Anopheles*

Enzyme	Honey bee	*Drosophila*	*Anopheles*
i-type lysozyme	1	4	2
c-type lysozyme	2	12	11
cellulase (endo-beta-1, 4-glucanase)	1	0	0
alpha(1,2) fucosyltransferase	1	0	0
dTDP-4-dehydrorhamnose 3,5-epimerase	1	0	2
N-acetylneuraminic acid phosphate synthase	0	4	2

### The cellulase gene

Cellulase catalyses the hydrolysis of internal beta (1,4) glycoside bonds in cellulose and is found mainly in plants, bacteria and fungi (Tomme *et al*., 1995). The first cellulase gene in animals was identified in termites and is a member of the GHF9 family of glycoside hydrolases (Watanabe *et al*., 1998). GHF9 genes have now been described in several animal species distributed sporadically across taxa (termites: Tokuda *et al*., 1999; cockroaches: Lo *et al.*, 2003; crayfish: Byrne *et al*., 1999; sea squirts: Dehal *et al*., 2002; abalone: Suzuki *et al*., 2003). Because cellulase is missing in many species, including *Drosophila* and *Anopheles,* it was originally thought that endogenous cellulase activity in animals arose by horizontal gene transfer, but more recent evidence suggests that GHF9 genes in metazoans arose from a common ancestor (Davison & Blaxter, 2005). A honey bee cellulase gene was identified in an earlier version of the *Apis* genome as part of a large phylogenetic study (Davison & Blaxter, 2005). Here, we further characterize this gene.

**Figure 2:**
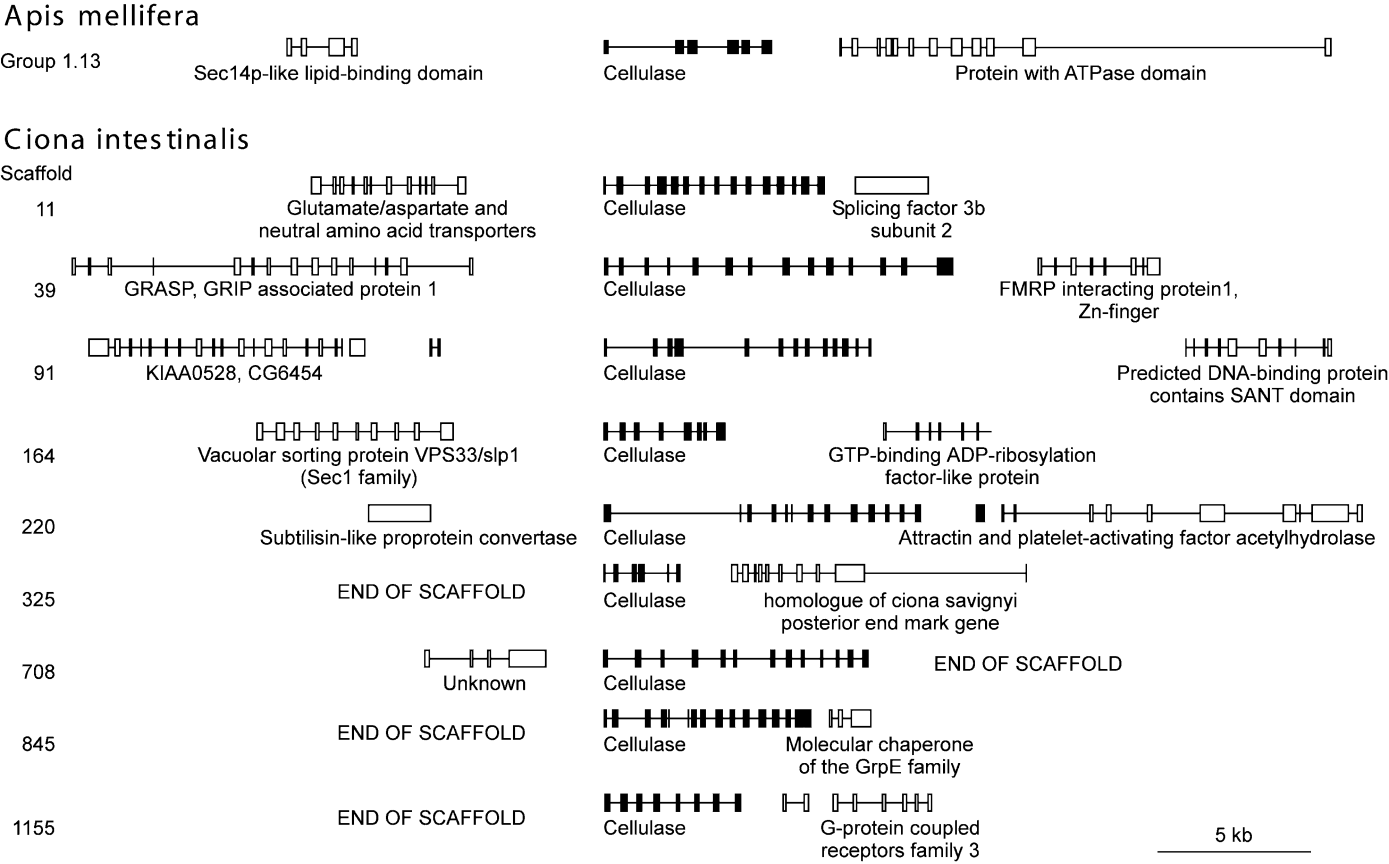
Comparison of the neighbouring genes of cellulase in the genomes of *Apis mellifera* and *Ciona intestinalis*. Location and structure of genes neighbouring the cellulase gene are shown. Boxes and connecting lines represent exons and introns. Cellulase genes are shown in the centre (black boxes). There is a single cellulase gene in the *Apis* genome, whereas the *Ciona* genome contains 11 putative cellulase genes. Scaffolds 5649 and 7316 of *Ciona* contain no predicted genes other than cellulase and are omitted from the figure. In four cases, a cellulase gene is located at the end of a scaffold in the *Ciona* genome, and neighbouring genes cannot be identified.

We found one GHF9 (cellulase) gene (GB13443) in the latest assembly of honey bee genome, which shows striking similarities with termite endo-beta 1, 4-glucanases. To exclude the possibility of bacterial contaminant, we examined its precise gene structure. This gene is located in Group 1.13 and contains at least five introns surrounded by genes that are clearly endogenous to the honey bee – protein with Sec14p-like lipid-binding domain (GB16852) and protein with ATPase domain. These results confirm that the cellulase gene is of honey bee origin. The sea squirt, *Ciona intestinalis,* also possesses cellulase genes and its whole genome has been analysed (Dehal *et al*., 2002). We compared the orders of the genes that are flanked 5′-upstream and 3′-downstream of the *Apis* cellulase and eleven *Ciona* cellulase genes, but we found no conservation of these gene orders (Fig. 2). Although we also examined the possibility that the cellulase gene alone dropped out and the flanking genes remained in the same genome location, the order of the two genes that flank the honey bee cellulase gene is not conserved in the *Drosophila*, *Anopheles* and *Caenorhabditis elegans* genomes, all of which lack cellulase genes (data not shown). Therefore, it is likely that extensive genome reorganization has occurred near the cellulase gene over the course of insect evolution. Furthermore, the persistence of cellulase in the bee genome cannot be explained by selection to preserve surrounding genes, because synteny in this region has not been conserved among insects.

Wood-eating insects such as termites or cockroaches presumably require cellulases to digest their cellulose-rich foods. In contrast, nectar and pollen, the sole dietary components of the honey bee, are not high in cellulose. Because of high structural similarity to the termite GHF9 gene, we suggest that the honey bee cellulase functions as an endo-cellulase. The wall of pollen grains can be subdivided to two layers and the inner wall consists mainly of cellulose (Knox & Heslop-Harrison, 1970). Thus, cellulase may be needed to digest the cellulose wall to release all the nutrients from the pollen and to digest cellulose contaminants in the nectar. Indeed, the available ESTs and microarray expression data strongly support this notion. We found a number of cellulase ESTs in two libraries obtained from heads/brains of adult bees (BI515449 and other ESTs, BeeBase web site http://racerx00.tamu.edu/bee_resources.html). Both the quantity of the available ESTs and their level of expression in the head suggest that this gene belongs to a class of highly abundant gland genes and is most likely associated with the hypopharyngeal (HP) gland (Whitfield *et al*., 2002). If so, this would expand the known repertoire of hydrolytic enzymes produced by the HP gland and suggest that honey bees might be capable of digesting the inner wall of pollen grains. Another possibility is that cellulase may function in the production of propolis by digesting other plant materials containing cellulose.

### Glucose oxidase/dehydrogenase genes

Glucose dehydrogenase (acceptor) catalyses the reaction of beta-D-glucose and acceptor to D-gluconolactone (D-gluconic acid) and a reduced acceptor (Bak, 1967) and is found in various organisms. Glucose oxidases catalyse the conversion of beta-D-glucose and O_2_ to D-gluconolactone (D-gluconic acid) and H_2_O_2_ (Bentley, 1963) and is quite rare in the animal kingdom. Glucose oxidases confer antiseptic activity to honey by producing D-gluconic acid and H_2_O_2_. Ohashi *et al*. (1999) identified glucose oxidase activity from homogenates of forager hypopharyngeal glands, sequenced the protein's fourteen most N-terminal amino acid residues, and used this sequence to isolate a cDNA for *Apis* glucose oxidase.

**Figure 3:**
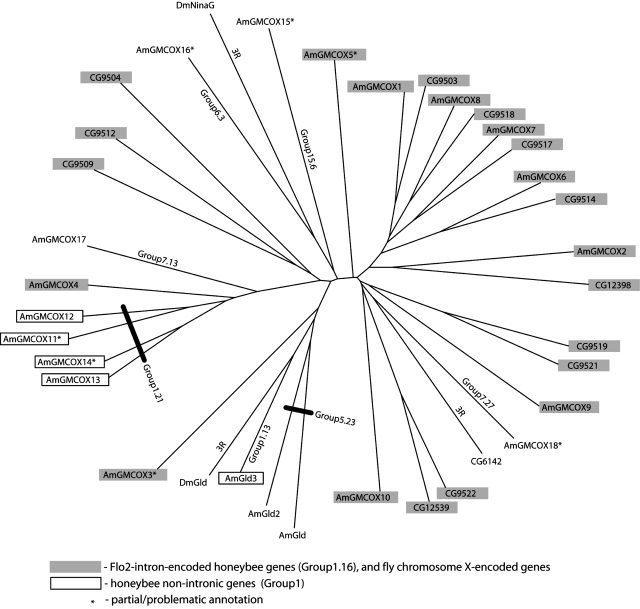
Phylogenetic tree of glucose-methanol-choline (GMC) oxidoreductases of both *Drosophila* and the honey bee. Honey bee glucose oxidases (*Amglox1–2*) show the closest relationship with *Drosophila* glucose dehydrogenase (*Dmgld*) and the honey bee gene *Amgld. Flo-2*-intron-encoded genes (bee Group1.16 and fly chrom.X) are highlighted in grey. Non-intronic genes (bee Group1) are in boxes. * denotes partial annotations.

We found a total of three glucose dehydrogenase/glucose oxidase genes in the *Apis* genome. The previously identified glucose oxidase is encoded by *Amglox1* (GB19418) and is located adjacent to a second glucose dehydrogenase gene, *Amglox2* (=*Amgld2;* GB13065) on Group 5.23. These two genes are not only phylogenetically related (Fig. 3) and closely linked on Group 5.23, but also show a similar pattern of expression in the head consistent with HP gland expression (ESTs and microarray data, Whitfield *et al*., 2002). The third glucose dehydrogenase gene, *Amgld1* (GB20157), is located on Group 1.13 (Fig. 3). There is only one closely related fly gene, *Dmgld* (CG1152). Phylogenetic analysis suggested that *Amgld1* is the orthologue of *Dmgld*, while *Amglox1* and *Amglox2* are close paralogues (Fig. 3). All these genes are more distantly related to GMC oxidoreductases. We speculate that glucose oxidase evolved by two steps. First, a gene duplication event produced the tandem glucose dehydrogenase genes *Amglox1* and *Amglox2* from an ancestral GMC oxidoreductase gene. Subsequent changes in the *Amglox1* gene led to its conversion from a glucose dehydrogenase to a glucose oxidase. Of course, it is possible that any of the other glucose dehydrogenases (including *Dmgld*) may also function as glucose oxidase, so the enzymatic activity of all these enzymes should be studied.

This is the first glucose oxidase gene to be found in an animal genome. Until other putative glucose dehydrogenases are tested for their ability to catalyse the glucose oxidase reaction, it is impossible to know how rare glucose oxidases actually are among the insects. Nonetheless, this gene provides valuable clues to the evolution of glucose oxidase enzymes and to enzyme evolution in general, as the conversion of substrate specificity is thought to be a common means by which new enzymatic functions evolve. Along with the parallel case of glucose-6-phosphatase (above), glucose oxidase demonstrates that comparison of honey bee and *Drosophila* genomes will be a powerful tool with which to identify and study recently evolved enzymes in the insects.

### GMC oxidoreductase genes of the honey bee and Drosophila

GMC oxidoreductases are FAD flavoproteins with diverse but poorly understood catalytic activities. Several GMC oxidoreductases have been implicated in specific physiological processes such as sperm storage, cuticle biosynthesis and glucose metabolism. We identified twenty-one members of this family in *Apis* compared to sixteen in *Drosophila*. The increase in the size of this family is largely due to four new members (AmGMCOX11-14) in Group 1.21 forming a closely related branch that apparently evolved from AmGMCOX4 (Fig. 3).

**Figure 4:**
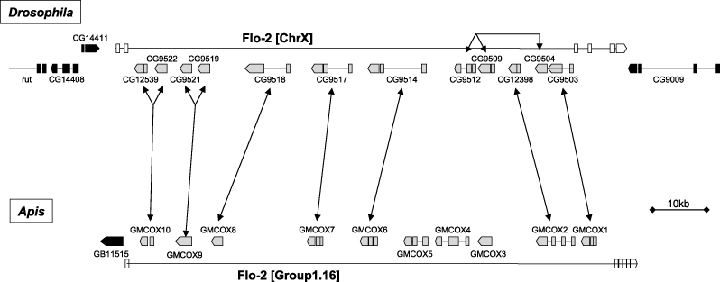
Glucose-methanol-choline (GMC) oxidoreductase gene synteny for the honey bee and *Drosophila*. Most of the GMC oxidoreductases form a cluster and their order in the genome is highly conserved between the honey bee and *Drosophila*. This cluster resides in the intron of the *Flo-2* gene encoded on antisense strand. The *Flo-2* gene and other genes are shown in white and black, respectively. Boxes and connecting lines represent exons and introns.

In both species, ten to twelve of the GMC genes cluster together, and the organization of the genes within the cluster is well conserved. Remarkably, all ten clustered GMC oxidoreductase genes in the bee are encoded within a large 90 kb intron of the *flotillin-2* gene in Group 1.16. These intronic genes are encoded on the DNA strand opposite *flotillin-2*. Seven GMC oxidoreductases are themselves interrupted by introns; the remaining three appear to be intronless (Fig. 4). This model is supported by ESTs (BeeBase). Flotillin-1 and -2 are markers for membrane microdomains (lipid ‘rafts’), and they participate in growth factor-induced regulation of the actin cytoskeleton. The functional relationship between flotillins and GMC oxidoreductases is not known. This nested gene architecture of GMC oxidoreductases is remarkably well-conserved in *D. melanogaster* (Fig. 4) and in the beetle *Tribolium castaneum* (not shown). One large intron of *Drosophila flotillin-2* contains twelve genes on the opposite strand encoding both intronless and intron-containing transcription units.

One explanation for the unusual stability of this genomic landscape is that these gene structures are so intricately organized that events that break them apart are very rare. The interesting question is whether they are ‘trapped’ in this configuration because of the low likelihood of producing a rearrangement that can separate the components, or because of higher order regulation of the nested genes. Given the fact that these genes encode enzymes with similar or even identical catalytic activities, a common regulatory control might be advantageous for their effective expression.

### Fucosyltransferases

Fucosyltransferases (FUTs) catalyse the transfer of fucose in alpha(1,2), alpha(1,3/4) or alpha(1,6) linkages on various glycans (Javaud *et al*., 2003). Alpha(1,2) FUT adds fucose in alpha(1,2) to the terminal galactose residue of lactosamine to make blood-type H antigens in humans. Alpha(1,3/4) FUT adds fucose in alpha(1,3) or alpha(1,4) to the GlcNAc residue of lactosamine. Alpha(1,6) FUT adds fucose in alpha(1,6) to the core GlcNAc residue in N-glycans. Phopholipase A2, a major component of honey bee venom, is alpha(1,3)- and alpha(1,6)-fucosylated (Kubelka *et al*., 1993).

**Figure 5:**
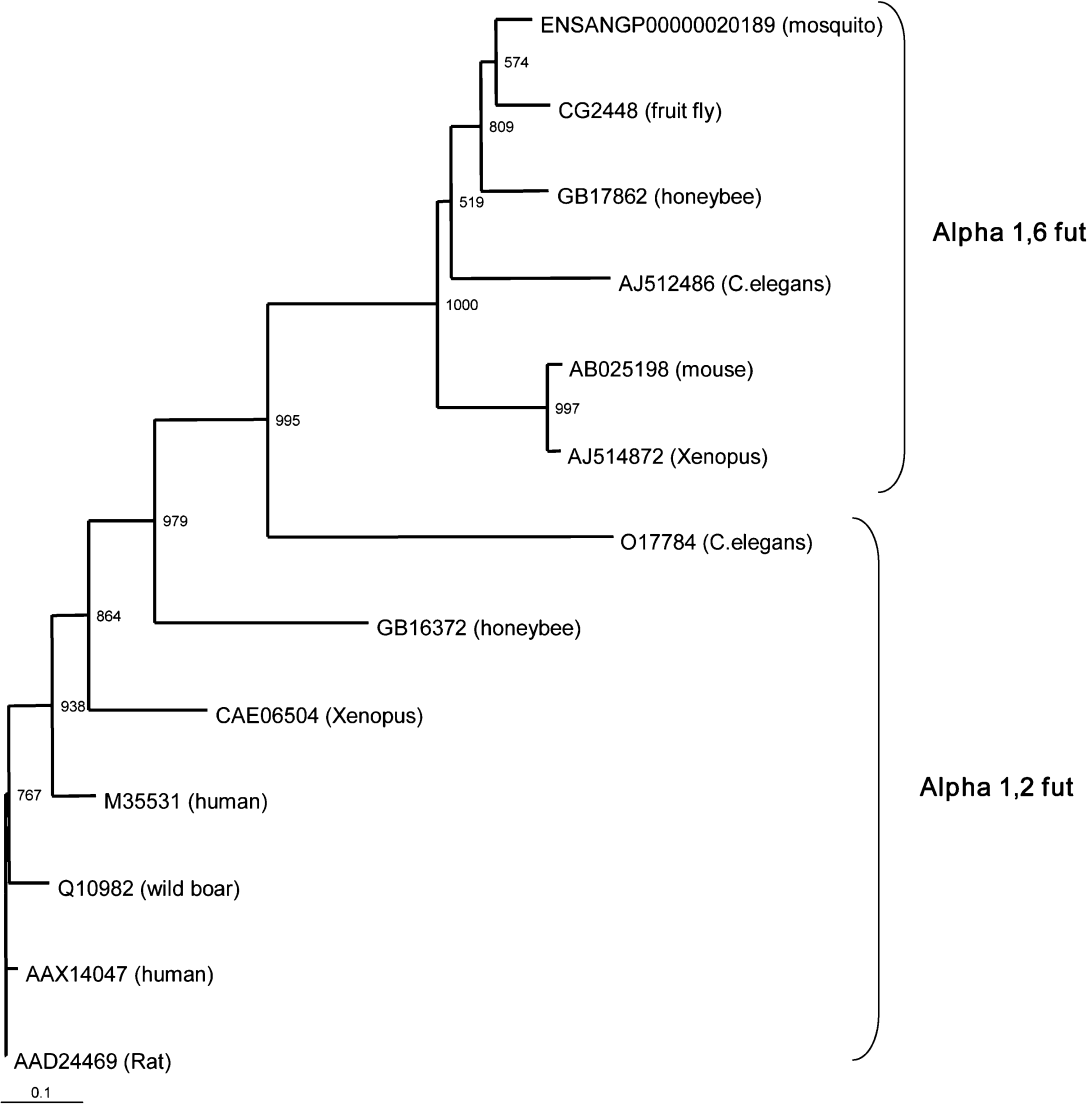
Phylogenetic tree made with the selected 54–55 amino acid residues comprising the three conserved motifs (I, II, III) of α (1,2) and α (1,6) FUTs using the neighbor-joining method. Numbers at each node represent bootstrap values as a percentage of 1000 trials.

We identified a single alpha(1,6) FUT gene in each of the three insect genomes and 4 : 4 : 2 alpha(1,3/4) FUT genes. Phylogenetic analysis suggests that the genes for alpha(1,6) FUT (Fig. 5) as well as alpha(1,3/4) FUT are conserved among honey bees, flies, mosquitoes, nematodes and vertebrates (Fig. 5). However, we could not identify any alpha(1,2) FUT genes in the dipteran genomes, in agreement with previous findings for *Drosophila* (Roos *et al*., 2002). Using an alpha(1,2) FUT sequence from wild boar, *Sus scrofa*, we identified a single alpha(1,2) FUT gene in the bee.

Alpha(1,2) FUTs are common in many taxa, so the absence of this enzyme in dipterans is most easily explained as a gene loss. The presence of alpha(1,2) FUT in the bee indicates that the gene loss is confined to a subset of insect species. Mammalian FUTs are involved in determining blood type and in defence against microbe infection (Hurd & Domino, 2004;Iwamori & Domino, 2004), so *Apis* alpha(1,2) FUT may also have roles in the immune system. Although the fucosyl groups on phospholipase A2 have alpha(1,3) and alpha(1,6) linkages rather than an alpha(1,2) linkage, it remains possible that this enzyme is involved in the modification of venom components.

### Lysozymes

Lysozymes break alpha(1,4) linkages between N-acetylmuramic acid and N-acetylglucosamine of bacterial peptidoglycan (Jolles & Jolles, 1984) and are thought to play roles in defence against bacterial infection (Ellison & Giehl, 1991; Hultmark, 1996). Two principal classes of lysozymes are c-type (chicken) and i-type (invertebrate), both of which are found in dipteran genomes (Ito *et al*., 1999; Bachali *et al*., 2002).

**Figure 6:**
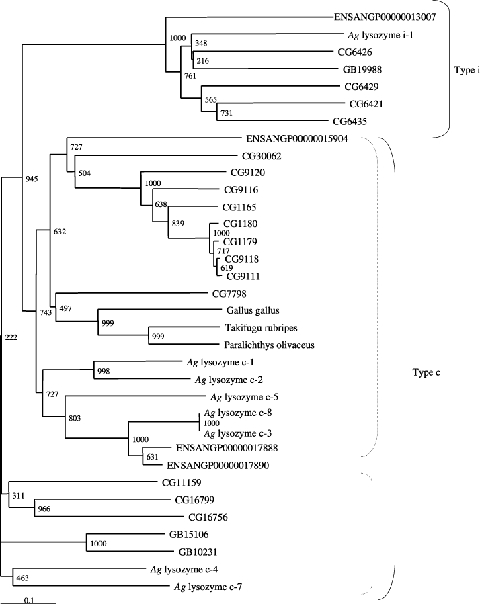
Phylogenetic tree showing i- and c-type lysozymes using the neighbor-joining method. Numbers at each node represent bootstrap values as a percentage of 1000 trials. Dotted lines in the group of type c lysozymes indicate putative subgroup of type c lysozyme genes. Ag lysozyme c-6 was not included because it contains five lysozyme motifs.

**Figure 7:**
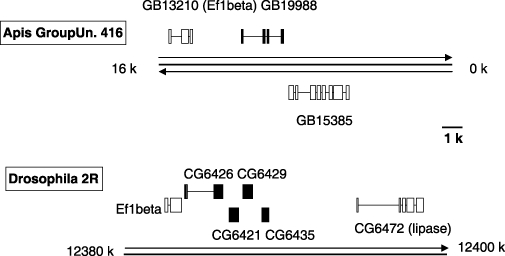
Comparison of the genome region surrounding the i-type lysozyme genes in *Apis mellifera* and *Drosophila melanogaster* (closed boxes). The genes surrounding the lysozyme genes are shown in open boxes. Boxes and lines show the exon-intron structure of each gene. Thick lines indicate the partial chromosomes and arrows indicate the direction of the genes.

We found 1 : 4 : 2 i-type lysozymes and 2 : 12 : 11 c-type lysozymes (Fig. 6). Gene mapping indicates conserved syntenic organization around the lysozyme genes: i-type genes are adjacent to EF1beta in both the bee and the fly (Fig. 7), and c-type genes are adjacent to each other in both species (data not shown). These results suggest that gene number differed dramatically after c- and i-type lysozymes separated in the course of insect evolution. We also noticed that the c-type lysozymes can be subdivided into two types with 0 : 9 : 8 and 2 : 3 : 2 genes in the three genomes (Fig. 6). The main sources of nutrients for *Drosophila* are the fungi and bacteria present in rotting fruit, and lysozymes are digestive enzymes that break down the cell walls of these microorganisms (Daffre *et al*., 1994; Regel *et al*., 1998). Thus, it is also plausible that the lysozyme gene family expanded in *Drosophila* as an adaptation to its feeding habits.

## Conclusions

Although much is known about individual metabolic enzymes, metabolomics and genome-wide analyses of metabolic enzymes are still in their infancy. The availability of multiple high-quality genome sequences from insects in species with divergent but well-defined feeding habits provides an opportunity to systemically explore the effects of environmental specialization on genomes. We characterized the genes encoding 202 carbohydrate- and lipid-metabolizing enzymes in the honey bee, by determining orthologous relationships with *Drosophila* and *Anopheles*. By using a pathway approach, in which each enzyme is viewed as part of larger, interconnected metabolic network, we were able to identify potential ‘hotspots of evolutionary change’ in metabolism.

Overall, there was a high degree of similarity at the levels of amino acid sequence and gene number. However, our data suggest differences in the rate of change across insect species for different pathways, at least at the level of gene number. Our results suggest gene number may be more dissimilar between insect species for glycolysis/gluconeogenesis than for several groups of lipid-metabolism enzymes.

For enzymes with established roles in bee biology we can say more. Unlike most insects, honey bees hoard food for long periods of time. This is adaptive for a species totally reliant on flowers, a highly ephemeral resource. In many regions of the world, flowers are available on a highly intermittent basis (Seeley, 1995). Nectar contains 15–40% sugar, and as such is particularly susceptible to bacterial degradation. Bees have evolved a suite of adaptations to be able to survive year-round on this source of food. These adaptations involve the conversion of nectar into honey, with physical and chemical properties that contribute to its long-term stability. By evaporating moisture from the nectar with a specialized behaviour (fanning), they convert nectar into honey, a supersaturated solution, with high osmotic potential, making it difficult for bacteria to survive in it (Crane, 1996). Bees also add glucose oxidase to honey to produce hydrogen peroxide, a well-known antibiotic. Our analysis suggests that one challenge of bee feeding ecology was solved in part by the invention of glucose oxidase through a conversion in the enzymatic activity of glucose dehydrogenases.

Interestingly, the honey bees may have also solved the problem of extracting nutrients from pollen grains that are their primary protein source. Contrary to previous beliefs that bees do not have the enzymes needed to digest the complex carbohydrates (Grogan & Hunt, 1979) we report that a transcribed gene encoding cellulase is encoded by the genome of this species. We interpret this result as strongly supportive of the idea that bees are capable of removing the cellulose layer from the pollen wall, hence improving the effectiveness of the digestive processes.

Conversion of enzymes to new substrate specificities may be an important mechanism by which organisms become specialized for their food habitats, and we hope that further comparison of insect genomes will identify more examples for further study. Similarly, cellulase, FUTs and lysozyme are probably responsible for digestion of cellulose (Nakashima *et al*., 2002), fucosylation of proteins or polysaccharides (Kubelka *et al*., 1993; Javaud *et al*., 2003), and breakdown of bacterial cell walls (Ellison & Giehl, 1991; Hultmark, 1996), respectively. The possible adaptive significance of the change in the numbers of the genes for these enzymes should be studied in future research.

Our pathway approach represents a starting point for showing how genome sequences can be used to understand changes in metabolism that may have accompanied the dietary specializations and energy requirements of animals with different evolutionary and life histories. We suggest that coupling this with a characterization of functional differences in metabolic pathways, including differences in metabolic gene expression and metabolite profiles, will be a promising postgenomic approach to understanding adaptive changes in metabolism.

## Experimental procedures

### Characterization of genes involved in lipid metabolism and synthesis, ketone body degradation and synthesis, and glycolysis/gluconeogenesis

We first identified metabolic enzymes involved in pathways of interest using databases of metabolic pathways linked to *Drosophila melanogaster* genes (KEGG; http://theseed.uchicago.edu/FIG/index.cgi). Using sequences from *Drosophila* at Flybase (http://flybase.net/) and *Anopheles gambiae* (accessed at NCBI; http://www.ncbi.nlm.nih.gov/), we searched the *Apis mellifera* genome Assembly 2 (BeeBase; http://racerx00.tamu.edu/cgi-bin/gbrowse/) for homologous sequences using TBLASTN searches. We confirmed hits by best reciprocal match and CLUSTALW alignments using at least two insect sequences and at least two vertebrate sequences. When necessary, we built genes manually using a combination of alignment tools and splice prediction algorithms (Flybase). A few enzymes appear to be completely missing from the fly and/or mosquito genomes. To analyse these genes, sequence from other insects or relevant species was used; termite cellulase (AB013272, *Nasutitermes takasagoensis*) and alpha(1,2) FUT of *Sus scrofa* (Q10982).

### Comparison of genome organization around genes of interest

Predictions for neighbouring genes were obtained by using Blast and consecutive genome browsing with the BeeBase genome browser assembly v 2.0. Similar processes were used for other species: FlyBase for *Drosophila*; Ciona database (http://genome.jgi-psf.org/ciona4) for *Ciona intestinalis*, and Ensemble (http://www.ensembl.org/) for *Anopheles* and *Caenorhabditis,* and conserved domains detected by BLASTP at NCBI.

### Phylogenetic analysis

Conserved regions/motifs were searched and characterized by CLUSTALW. Phylogenetic analyses were performed with single conserved motif or combined sequence constituted by the juxtaposition of multiple conserved motifs. Phylogenetic trees were made with the neighbor-joining method. The statistical significance of branch order was estimated by performing 1000 replications of bootstrap re-sampling of the original aligned amino acid sequences. For fucosyltransferase, CLUSTALW revealed three conserved peptide motifs I, II, III and juxtaposition of these three conserved motifs resulted in 54 and 55 amino acid residues which were used for phylogenetic analysis (Martinez-Duncker *et al*., 2003). For motif comparison, the following genes were used; alpha(1,2) FUT of *Sus scrofa*; Q10982, *Caenorhabditis elegans*; 017784, *Xenopus laevis*; CAE06504, *Homo sapiens*; M35531 and AAX14047, *Rattus norvegicus*; AAD24469, and α (1,6) FUT of *Caenorhabditis elegans*; AAN84870, *Mus musculus*; AB025198, *Xenopus laevis*; AJ514892, *Drosophila melanogaster*; AY01451 and *Anopheles gambiae*; ENSANGP00000020189. The conserved motifs of GB16372 and GB17862 were I(206–218), II(249–263) and III(294–310) for GB16372, and I(369–381), II(412–425) and III(460–485) for GB17862.

## Supplementary Material

Table S1 Gene list of carbohydrate metabolizing enzymes analysed in the broader survey. In total, 101 enzymes are shown and these enzymes are categorized by their substrate (32 major compounds and others). Corresponding honeybee genes are also shown in the official gene prediction ID (total 148). This material is available as part of the online article from http://www.blackwell-synergy.com.
